# Surviving the COVID‐19 pandemic: The antecedents of success among European SMEs

**DOI:** 10.1111/emre.12525

**Published:** 2022-05-23

**Authors:** Myint Moe Chit, Richard Croucher, Marian Rizov

**Affiliations:** ^1^ Nottingham University Business School University of Nottingham Malaysia Semenyih Malaysia; ^2^ Middlesex University Business School Middlesex University Hendon UK; ^3^ Lincoln International Business School University of Lincoln Lincoln

**Keywords:** COVID‐19, enterprise survival, government support, innovativeness, SMEs, World Bank Enterprise Survey

## Abstract

We research the antecedents of relative success among small and medium enterprises (SMEs) in avoiding temporary or permanent closure during the COVID‐19 pandemic. We investigate the roles of firm‐specific resources and state support policies in influencing SME fortunes, in a sizeable group of European countries covered in the World Bank Enterprise Survey. Using resource dependency, Varieties of Capitalism and Systems theories, we find that innovative capacities, institutional connectedness, governance, and management experience were major antecedents of success across all SMEs. Significant differences in outcomes were found between SMEs operating in old and new EU member states, and non‐EU countries.

## INTRODUCTION

We research the antecedents of relative success in avoiding the worst effects of the COVID‐19 pandemic (temporary suspension or reduction of activity and permanent closure) among small and medium enterprises (SMEs). We investigate the respective roles of firm‐specific resources and state support policies in influencing SME fortunes, in a sizeable group of European countries included in the World Bank Enterprise Survey (WBES). Previous research is predominantly at the national level.

Our investigation builds on considerable previous work in this journal dealing with managerial problems in organizations arising from crisis, such as those included in a virtual special edition on the COVID crisis (April 2020). Several cognate articles have adopted an organizational‐psychology viewpoint and complement our approach. Chatrakul Na Ayudhya et al. (2019) are concerned with the nature of impacts on leaders and employees of the 2008 Global Financial Crisis in Greece where SMEs feature strongly. Mahmoud et al. (2021) deal with individual employees' psychological reactions to COVID‐19 in a large SME setting in Middle East and North‐African countries. In common with these works, we also examine an issue of great importance to both leaders and employees, namely the impact of COVID‐19 on employment in small companies and the fundamental question of the antecedents of success and failure in enterprises surviving that shock. We replicate previous works' interest in leadership and also introduce a range of other, theoretically derived organizational considerations in numerous national settings across different European regions.

**FIGURE 1:**
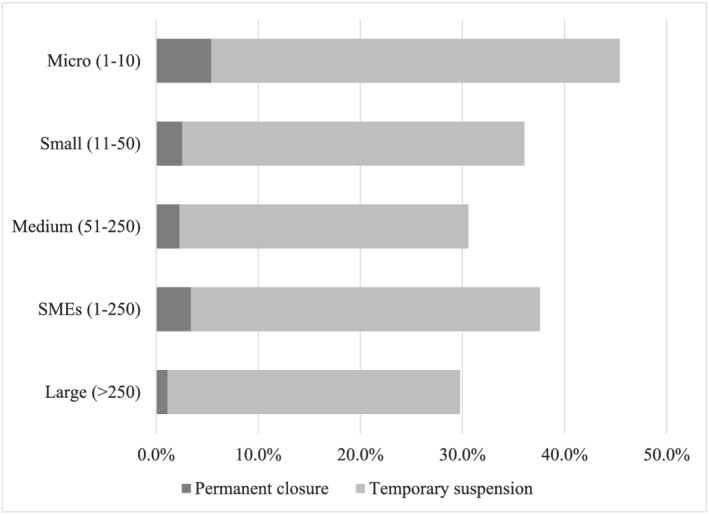
The proportion of firms closure by size category. Source: Authors calculation based on WBES COVID‐19 follow‐up surveys

Following the January 2020 outbreak of COVID‐19, many businesses in Europe suspended or terminated their operations. The OECD (2020) reported that the pandemic's effects on SMEs were especially severe due to their vulnerability to shocks. Around 4% of European SMEs reported that they closed permanently and 37% temporarily closed by suspending provision of services or production (see Figure 1). Micro‐enterprises were especially negatively affected. In contrast, only about 1% of large firms have permanently closed and less than 30% reported temporary closure. To mitigate adverse impacts, the EU and national governments provided support plans and stimulus packages, intended to address cash flow issues, support wages and incomes of suspended employees, and give fiscal exemptions such as tax deferrals and debt payment holidays (OECD, 2020).

Managers and policy makers should understand the characteristics of those SMEs that were relatively (un)successful in avoiding the crisis' worst effects. Literature on SMEs provides little guidance on organizational sustainability in general; cross‐company studies tend to treat SMEs simply as “scaled down” large companies (Darcy et al., 2014:379). Few works have dealt with SME issues in relation to COVID‐19 or previous rather different shocks such as the 2008 financial crisis (Doern et al., 2019; Herbane, 2010; Wenzel et al., 2021). Although some excellent studies of the more recent COVID‐19 shock have appeared, most report on specific countries—Brown and Cowling (2021) on the UK and Adam and Alarifi (2021) on Saudi Arabia. They stress the importance of strong financial reserves (Brown & Cowling, 2021) and innovation (Adam & Alarifi, 2021) to sustainability. The generalizability of their findings to other countries is unclear.

We use the recent and extensive WBES on the impact of COVID‐19 in the private sector, allowing generalization across countries. We deploy resource dependency and institutional theories, addressing *en route* differing expert views on the effectiveness of institutions in different parts of Europe. We focus on three related questions. First, which SMEs have been most severely affected? Second, which characteristics are most relevant to relative success? Third, what has the overall impact of government support policies been?

Countries covered by WBES include larger countries such as Russia, several Mediterranean nations (e.g., Italy, Greece, and Cyprus), and Central and East European post‐socialist countries (e.g., Hungary, Slovenia, and Moldova). Both established EU member states and “new entrant” countries are therefore represented. The database does not cover all European countries, and notably omits important West European countries, but has considerable coverage of contrasting economies within which SMEs play quite different roles, and whose governments have differing relationships to the EU.

Our findings highlight some interesting characteristics of relatively successful SMEs. They nuance recent criticisms of institutional functioning in post‐socialist economies (e.g., Kadriu et al., 2019). We find that government support measures have generally been beneficial to SMEs during the crisis—their effects are often conditional on the prevailing financial and institutional conditions. The support‐measure effects are also clearly linked to the wider containment policy packages adopted by different countries and the rigor of their implementation.

The paper is structured as follows. Section 2 discusses theories on the role of firm‐specific resources, institutions, and strategies in mitigating the adverse impacts of crisis and develops testable hypotheses. Section 3 provides an overview of the data and empirical methods used. Section 4 reports the results of our empirical analyses, while section 5 concludes.

## THEORY AND HYPOTHESES

We test two bodies of theory as lenses through which to examine the impact of COVID‐19 on SMEs' survival: resource dependency and institutional theory. Within the latter, two variants of institutional theory are deployed: Business Systems and Varieties of Capitalism. These theories were all initially developed outside of the “transitional” economies which play a considerable role in our dataset. “Transitional” environments have been identified as posing different challenges to SME survival from those within long‐established capitalist economies (Kadriu et al., 2019). Hence, our central bodies of theory must be tested with sensitivity to that argument.

### Innovation and survival

Resource Dependency Theory (RDT) suggests that SME survival depends on companies' capacity to deal with external shocks (Pfeffer & Salancik, 1978). RDT has evolved to prescribe innovation as a means to that end (Hillman et al., 2009). RDT has commonly been used in connection with SMEs, mainly because the latter's limited internal resources, particularly in “transitional” (and emerging) countries, increase their reliance on external networks and institutions to secure their survival (Pissarides, 1999). RDT is related to the Resource Based View (RBV) of the firm, but the emphasis within RDT is on firm survival and resilience in the face of external shocks and is therefore useful in the COVID‐19 context. RBV is more concerned with firm competitive advantage.

RDT has been demonstrated to have greater explanatory power when deployed in tandem with institutional theory (Sherer & Lee, 2002). Both sets of theory recognize the significance of how managerial decisions are shaped by external contexts. Under “normal” conditions, satisfying the requirements of RDT permits firms to move on to satisfy those of RBV. Pfeffer and Salancik (1978), in their original formulation of RDT, proposed a range of external and related internal measures to maximize survival chances, namely, mergers and vertical integration, joint ventures and alternative inter‐organizational relationships, boards of directors, political action, and executive succession. We consider these in our discussion in the next two sections.

According to RDT, existing resource constraints may be managed through product and service innovation since these increase both resilience in the face of external shocks, and also the discovery and utilization of new resources (Hillman et al., 2009). Innovation is a collective, collaborative process promoted firstly by investments in employee training and secondly by investments in company research and development (Von Stamm, 2003). The former encourages employees to invest in their own development which in turn results in mutual long‐term expectations for employment relationships, in high levels of “employer‐employee interdependence” and low levels of employee turnover (Whitley, 2000). These are underlying preconditions for innovation of both the radical and incremental types. Despite their different requirements in other respects, both innovation types require highly involved employees (Von Stamm, 2003:271). Research and development investment complements investments in and by employees and is an indicator of the importance placed by companies on development of their products and services. Indeed, the capacity for rapid innovation has already been shown to be important to SMEs in surviving COVID‐19 (Adam & Alarifi, 2021).

Our data permit examination of the innovation variable. We therefore hypothesize:

SMEs with strong innovative capacities are more likely to have avoided closure.

### Institutional connectedness and survival

Institutional theory, in common with RDT, fundamentally rests on the notion that companies follow other similar companies in seeking legitimacy. Overlaps exist between the two theories. Thus, as RDT recommends, when advocating “alternative inter‐organizational relationships,” SMEs may create strong links to other companies. Such links are also noted as positive by one variant of institutional theory—Business Systems theory (Whitley, 2000)—as “employer‐employer” cooperative links, capable of supporting cross‐employer training. Moreover, unlike some other measures recommended by RDT, such links potentially create inter‐company mutual gains rather than simply new dependencies. SMEs' search for legitimacy is conducted in both national and international contexts, and other companies are frequently used as intermediary allies when SMEs seek access to international markets (Hessels & Terjesen, 2010).

Access to financial resources has been a major issue for SMEs during the pandemic (Brown et al., 2020). In this connection, their key external relationship is likely to be with state apparatuses at national, regional, and local levels. In addition to gate keeping these all‐important funds, the state has widespread presence, overarching authority, and responsibility within national territories even if state administrative capacities vary between nations. Funds were disbursed by many states to assist SMEs during the pandemic, but so too have advice and information, and its quality, especially in the employment law area, has also been an important issue for managements (Hartigan et al., 2020). In the context of a need for rapid access to funds and advice, the significance of SMEs' links to governments through political activity, contacts, and networks is a priori evident. Companies in countries where governments have low administrative capacities may have higher incentives to link with other organizations to obtain and share information. They may seek legitimacy and assistance in dealing with state agencies by consulting employer and trade organizations, chambers of commerce, etc. (Osabutey & Croucher, 2018).

“Intermediate” institutions (i.e., those performing a linking role between the state and companies) such as employers' associations have been highlighted as vital by another variant of institutional theory—Varieties of Capitalism theory—as significant channels for solving issues of state‐company coordination (Culpepper, 2001). One of their functions is to provide channels through which government financial assistance and advice may be effectively transmitted to recipients (Culpepper, 2001). They potentially play a key role by brokering information flows between companies and governments to increase the effectiveness of state resource allocation and are likely to be most effective for SMEs in “transitional” economies (Culpepper, 2001:292–3). They are also highly relevant in the COVID‐19 case since the effective distribution of state assistance funds has required intensive contact between companies and public bodies. SMEs, and especially micro companies, are likely to face fundamental issues because of their limited resources to access the funds and establish their *bona fides*. Conversely, Adam and Alarifi (2021) found that institutional connectedness played a positive role in assisting the innovations made in Saudi Arabian SMEs during the COVID‐19 crisis.

“Intermediate” institutions of the type referred to above may also provide valuable advice and support to SMEs in relation to accessing funds and other assistance from private institutions such as banks, often perceived as extremely challenging for innovative SMEs to access. Indeed, many SMEs in Europe became discouraged from even applying for bank assistance for innovation, and it has been argued recently that they require more external information and assistance in this area (Brown et al., 2020:21).

We therefore hypothesize:

SMEs with high levels of institutional connectedness are more likely to have avoided closure.

### Governance, management experience, and survival

Both RDT and the variants of institutional theory used here address the subject of companies' *internal* organization and their connections to both competitiveness and survival. Varieties of Capitalism and Business Systems theories both stress strong, effective internal governance institutions as key elements of comparative advantage (Hall & Soskice, 2001; Whitley, 2000). As noted above, RDT advocates the establishment of Boards of Directors in SMEs as a means of exercising power inside and outside the company to those ends.

Boards assist companies by developing strategic approaches in relation to their environments, internal policies and practices, management succession, and contingency planning (Pfeffer & Salancik, 1978). Boards of Directors have already been found to raise the likelihood of SMEs having internal precautionary funds, shown to have been vital to SME survival in the COVID‐19 crisis (Brown & Cowling, 2021; Cowling et al., 2021).

SMEs, especially outside of advanced economies, are widely recognized as frequently lacking these internal institutions (Croucher et al., 2013; Pissarides, 1999). Their managements have also been viewed as challenged by environmental change, particularly outside of advanced capitalist economies where management expertise and experience has long been regarded as limited, especially in the HRM area (Barrett & Mayson, 2008). Where entrepreneurs may be highly suited to the early phases of organizational growth, they are widely acknowledged to require more corporate management skills in later phases (Ibid.). As Akehurst et al. (2009) conversely demonstrate, certain SMEs with experienced managers and sophisticated management philosophies have been able to develop employee commitment and thereby to involve them strongly in innovative and entrepreneurial directions. This has allowed them to “innovate and adapt in a creative way” (Ibid:280).

We therefore hypothesize:

SMEs with strong internal governance and experienced managements are more likely to have avoided closure.

### An alternative view, additional considerations, and control variables

Recently, an alternative view specifically pertinent to the “transitional” economies of Eastern Europe in relation to SMEs has emerged. Writing immediately prior to the COVID‐19 crisis, Kadriu et al. (2019) examined the impact of national institutions on SMEs innovative activities by researching SME respondents in a wide range of “transitional” economies. They found that far from stimulating innovation, respondents suggested that institutional factors such as laws, bureaucracy, and inefficiency could act to block SME innovative activities and those SMEs customarily used bribery to circumvent them. Hence, the SME respondents perceived that they were effectively taxed when they attempted innovation. However, this finding simply relays respondents' complaints. It also does not in any way exclude the possibility that institutional connectedness was important to SME survival during the COVID‐19 crisis and may even imply its greater importance than at other times. What the above discussion suggests is that institutional factors, specific to differing geographies and historic conditions (e.g., EU member states vs. non‐EU countries) could play an important moderating role in the effects of the factors subject to our three hypotheses stated earlier.

As a part of the institutional environment characteristics, a number of studies provide evidence that the financial system and specifically access to external finance are important factors for innovation and growth of SMEs in general (e.g., Beck et al., 2005; Raj & Sen, 2015), and for companies operating in developing and “transitional” economies in particular (Chit, 2018). According to Cowling et al. (2015) and Zubair et al. (2020), the role of financial resources is even more important in the survival and continuation of SMEs during crises. This is so because small companies' access to external finance is likely to be limited for several reasons. First, the crisis spillover to the financial sector limits supply, especially for SMEs (McGuinness & Hogan, 2016). Second, the pandemic‐specific crisis results in reduced access to entrepreneurial finance, requiring personal and relational interactions (Brown et al., 2020). Third, severe loss of demand and disruption to supply chains leads to shortage of working capital (Lu et al., 2020). Thus, access to finance is an important factor conditioning the effects anticipated in our three hypotheses as well as the impact of the government support obtained by firms. It is logical to expect that financially constrained firms will benefit more from access to funding provided by the various government support measures.

Like other studies, we control for firm size, age, and (female) ownership as proxies for firm‐specific resources and capabilities. Cowling et al. (2015) find that age and size are important factors for SMEs' survival during crises. Bartoloni et al. (2021) assert that greater skills and knowledge dependent on enterprise size and experience are essential to react and adapt to the increased uncertainty and complexity in the business environment during crisis. We control for female ownership on SMEs' survival because numerous studies point out that female‐owned SMEs are more likely to be financially constrained (e.g., Muravyev et al., 2009).

The impact of the pandemic is likely to differ in different parts of the economy. Compared to retail businesses, firms in the manufacturing and service sectors were more likely to close down during the COVID‐19 pandemic (Hacıoğlu‐Hoke et al., 2021). Therefore, we include in our empirical models manufacturing, services, and retail sector controls. Furthermore, the effects of COVID‐19 on industries will also depend on the support measures provided by national governments, for which we include a set of control indicators. Many EU countries implemented comprehensive packages where firm support measures were components of their containment strategies (OECD, 2020).

Hence, as suggested by Bosio et al. (2020) and Chen et al. (2020), and following on our preceding discussion, the impact of the pandemic on business activities is likely to differ by geo‐political clusters of European countries. We expect major differences between the EU and non‐EU countries, and between old and new EU member states. Therefore, besides the full sample analysis, we conduct analyses by three subsamples. We expect that the non‐EU “transitional” countries exhibit “transitional” features in a pure form, while the former “transitional” countries which became EU members have been significantly influenced by EU policies and thus represent a hybrid case.

## MATERIALS AND METHODS

### Data

The World Bank conducted follow‐up surveys to its WBES to obtain a snapshot of the impact of COVID‐19 on the private sector in 25 European countries (as of April 22, 2021) where WBES was recently conducted. The COVID‐19 follow‐up survey instrument measures changes in sales, employment, and input purchases as well as financial status, and asks questions about liquidity problems and policy measures implemented to ameliorate COVID‐19's impact. For this study, we construct our data by combining the COVID‐19 follow‐up surveys with relevant firm‐specific characteristics from the recent WBES. We use firm‐specific, unique identifications to achieve this.

Table 1 presents summary statistics on the severity of COVD‐19's impact and the availability of support in the sample countries. After removing the firms with missing observations, the sample number of SMEs is 9572. Among them, 329 firms (3.4%) were recorded as permanently closed due to the pandemic. Among the remaining 9243 firms, 3188 (34.5%) are currently temporarily closed or have suspended their operations due to the pandemic. The old‐EU subsample has the highest number of permanently closed firms (5.8%) while 45% and 55% of SMEs in the non‐EU subsample have suspended operations or reduced employment, respectively. The number of firms which received support varies widely across the sample countries. SMEs in the old‐EU subsample received almost twice the support received by non‐EU countries.

**TABLE 1:** Impact of COVID‐19 on business operations and support received by sample country

Country	EU status	Permanent closure	N1	Temporary closure	Employment decrease	Number of supports received	N2
Albania	Non‐EU	0.94%	318	65.71%	77.33%	0.64	315
Belarus	Non‐EU	4.52%	465	9.46%	34.58%	0.05	444
B and H	Non‐EU	3.45%	203	22.45%	34.85%	0.63	196
Bulgaria	New EU	6.72%	506	26.91%	36.52%	0.39	472
Croatia	New EU	2.88%	312	28.71%	26.26%	1.03	303
Cyprus	New EU	1.94%	155	51.97%	28.97%	1.36	152
Czech Republic	New EU	1.99%	351	22.09%	33.64%	0.70	344
Estonia	New EU	0.39%	254	17.79%	31.53%	0.56	253
Georgia	Non‐EU	2.33%	473	61.90%	62.64%	0.44	462
Greece	Old EU	0.41%	493	34.22%	47.84%	1.61	491
Hungary	New EU	1.84%	597	11.60%	35.36%	0.34	586
Italy	Old EU	7.99%	413	58.95%	37.12%	1.01	380
Latvia	New EU	1.60%	188	10.27%	46.21%	0.04	185
Lithuania	New EU	0.50%	199	45.96%	28.18%	1.08	198
Malta	New EU	1.62%	185	29.12%	47.35%	1.04	182
Moldova	Non‐EU	1.28%	234	53.25%	63.27%	0.02	231
Montenegro	Non‐EU	1.55%	129	31.50%	59.71%	0.88	127
N. Macedonia	Non‐EU	0.38%	260	34.75%	45.94%	0.69	259
Poland	New EU	3.27%	826	20.90%	33.36%	1.52	799
Portugal	Old EU	10.01%	739	26.77%	25.79%	0.57	665
Romania	New EU	3.53%	481	24.78%	31.56%	0.70	464
Russian	Non‐EU	4.17%	984	65.43%	61.36%	0.47	943
Serbia	Non‐EU	0.75%	266	18.94%	38.61%	1.67	264
Slovak Republic	New EU	4.13%	315	37.09%	35.62%	0.76	302
Slovenia	New EU	0.00%	226	35.40%	36.31%	0.93	226
Old EU		5.79%	1985	37.54%	35.53%	1.03	1870
New EU		2.89%	4,255	23.88%	33.95%	0.79	4,132
Non‐EU		2.73%	3,332	46.25%	54.32%	0.53	3,243
Total		3.44%	9,572	34.49%	40.70%	0.75	9,243

*Note*: N1 represents the number of observations after excluding firms with missing information. N2 represents the number of observations (N1) minus permanently closed firms (329).

### Variables

The dependent variables in our analysis are binary indicators: *Permanent Closure* ‐ the firm has permanently ceased operation, *Temporary Closure* ‐ the firm has temporarily suspended operation (production or service), and *Employment Decrease* ‐ worker numbers or working hours reductions are recorded relative to the same month in 2019.

To test our three theory‐based hypotheses, we construct three empirical explanatory variables (scales)—*Innovativeness*, *Institutional Connectedness*, and *Governance*—applying the Mokken nonparametric scaling model (Mokken & Lewis, 1982). The unweighted sum of item scores has to be monotonously related to the latent true scores which implies that the Mokken model provides estimates of the scale scores only at the ordinal level. As in other studies, the primary scaling criterion is Loevinger's H‐coefficient of homogeneity. A set of items constitutes a scale if the associated H‐coefficient exceeds 0.30. Cronbach's alpha measures a scale's reliability with an acceptability threshold of 0.50. The details of the items included in our empirical scales, results of the scaling procedure, and definitions of all other (control) variables used in our empirical analysis are reported in Table 2. Controls comprise enterprise‐specific characteristics such as management experience (associated with H3), financial constraints, (female) ownership, firm size and age, and industry indicators.

**TABLE 2:** Descriptions of variables

Variable	Description	Source
Permanent closure	Dummy variable = 1 if the firm is permanently closed.	WBES COVID follow‐up
Temporary closure	Dummy variable = 1 if the firm is or was temporarily closed (suspended services or production).	WBES COVID follow‐up
Employment decrease	Dummy variable = 1 if the total number of firms employees decreased or the firms total hours worked per week decreased in the last month compared with the same month in 2019.	WBES COVID follow‐up
Innovativeness	Ordinal variable constructed using Mokken scaling model (Loevinger H‐coefficient = 0.39, Cronbachs alpha = 0.7131). Component dummy variables: (1) the firm has introduced new or improved products or services; (2) the new or improved products or services are new for the firms main market; (3) the firm has introduced new or improved processes; (4) the firm has spent on the acquisition of external knowledge; (5) the firm has spent on own research and development activities; (6) the firm has spent on research and development activities contracted with others; (7) the firm has provided formal training programs for its permanent, full‐time employees; and (8) the firm is an exporter.	WBES
Institutional connectedness	Ordinal variable constructed using Mokken scaling model (Loevinger H‐coefficient = 0.32, Cronbachs alpha = 0.7420). Component dummy variables: (1) any of the members of top management has ever been elected or appointed to a political position, (2) the firm is part of a business support group, (3) information on government regulations obtained through the membership is useful, and (4) influencing regulatory decision‐making processes due to the connections is useful.	WBES
Governance	Ordinal variable constructed using Mokken scaling model (Loevinger H‐coefficient = 0.50, Cronbachs alpha = 0.5421). Component dummy variables: (1) the firm has formalized, written business strategy with clear key performance indicators, and (2) the firm has a board of directors or a supervisory board.	WBES
Management experience	Continuous variable = years of working experience in the sector by the top manager.	WBES
No financial constraint	Dummy variable = 1 if self‐reported access to finance is NOT an obstacle for the firms operation.	WBES
Female‐owned	Dummy variable = 1 if at least 50% of the firm is owned by a female.	WBES
Micro	Dummy variable = 1 if the number of full‐time employees is 10 or less.	WBES
Small	Dummy variable = 1 if the number of full‐time employees is 11–50.	WBES
Medium	Dummy variable = 1 if the number of full‐time employees is 51–250.	
Firm age	Continuous variable = years of operation.	WBES
Manufacturing	Dummy variable = 1 for manufacturing firm.	WBES
Retail	Dummy variable = 1 for retail firms.	WBES
other services	Dummy variable = 1 for service firms.	WBES
Number of supports	Continuous variable = total number of government support measures the firm obtained (or expected to obtain) since the outbreak of COVID‐19. Support measure dummy variables: (1) cash transfer, (2) deferral of credit payments, rent or mortgage, suspension of interest payments, or rollover of debt, (3) access to new credit, (4) fiscal exemptions or reductions, and (5) wage subsidies.	WBES COVID follow‐up

Institutional and geo‐political differences are taken into account by distinguishing between EU member states and European countries outside the EU. To investigate the effectiveness of government support policies in mitigating the impact of COVID‐19, we employ an indicator of the number of support instruments that the firm has benefited from out of a set of five available: cash transfer, deferral of credit re‐payment, new credit, fiscal exemption, and wage subsidy. Considering that obtaining government support is likely to depend on the needs and also the capabilities of the firm, we treat the government support variable as endogenous and use an instrumental variables technique in our estimations. Table 3 presents summary statistics of the variables used in regression analyses.

**TABLE 3:** Summary statistics

	All		Old‐EU		New‐EU		Non‐EU	
Variable	Mean	S.D.	Mean	S.D.	Mean	S.D.	Mean	S.D.
Permanent closure	0.034	0.182	0.058	0.234	0.029	0.168	0.027	0.163
Temporary closure	0.345	0.475	0.375	0.484	0.239	0.426	0.463	0.499
Employment decrease	0.520	0.500	0.472	0.499	0.458	0.498	0.627	0.484
Innovativeness	1.561	1.755	1.456	1.647	1.605	1.776	1.567	1.789
Institutional Connectedness	0.804	1.133	1.063	1.169	0.864	1.156	0.572	1.033
Governance	0.707	0.771	0.992	0.812	0.640	0.746	0.623	0.739
Management experience	21.537	11.360	27.588	12.587	21.698	10.507	17.727	9.955
No financial constraint	0.474	0.499	0.584	0.486	0.535	0.499	0.450	0.498
Female‐owned	0.222	0.415	0.189	0.392	0.240	0.427	0.218	0.413
Micro	0.317	0.465	0.347	0.476	0.325	0.468	0.291	0.454
Small	0.435	0.496	0.436	0.496	0.432	0.495	0.437	0.496
Medium	0.245	0.430	0.217	0.412	0.239	0.426	0.270	0.444
Firm age	20.092	13.753	27.028	19.266	20.088	10.925	15.966	11.147
Manufacturing	0.527	0.499	0.587	0.492	0.541	0.498	0.474	0.499
Retail	0.179	0.384	0.170	0.376	0.168	0.374	0.198	0.399
Other services	0.294	0.455	0.242	0.429	0.291	0.454	0.327	0.469
Number of supports	0.751	1.085	1.039	1.300	0.793	1.059	0.532	0.924

*Note*: For number of observations refer to Table 1.

### Estimation methodology

Our observed dependent variables are binary, taking the value 1 if the firm has been affected by the COVID‐19 shock, and 0 otherwise. Therefore, we use a Probit model for our estimations. To detect possible endogeneity problems, we introduce the explanatory variables stepwise in our estimations starting with the controls and adding the main explanatory variables next. Observing stable estimated coefficients and increasing explanatory power of the model as indicated by Adj.*R* ^2^ would suggest no serious endogeneity problems.

The baseline empirical model specification with the dependent variable *Permanent Closure* is formalized in Equation (1):

(1) $$E\left(\text{Permanent}\;{\text{Closure}}_{\mathrm{ij}}\right)=\begin{array}{l} \phi (\beta_{0}+\beta_{1}{\text{Innovation}}_{\mathrm{ij}}\\ +\;\beta_{2}{\text{Institutional Connectedness}}_{\mathrm{ij}}\\ +\;\beta_{3}{\text{Governance}}_{\mathrm{ij}}\\ +\;\beta_{4}\text{Management Experienc}e_{\mathrm{ij}}\\ +\;\beta_{5}\mathrm{No}\;\text{Financial Constrain}t_{\mathrm{ij}}\\ +\;\beta_{6}\text{Female}-\text{owne}d_{\mathrm{ij}}\\ +\;\beta_{7}{\text{Micro}}_{\mathrm{ij}}+\beta_{8}{\text{Small}}_{\mathrm{ij}}\\ +\;\beta_{9}\text{Firm}\;\mathrm{Ag}e_{\mathrm{ij}}+\beta_{10}\text{Retai}l_{\mathrm{ij}}\\ +\;\beta_{11}{\text{Services}}_{\mathrm{ij}}+\varepsilon_{\mathrm{ij}}), \end{array}$$

where φ is a Probit function, *ε* is a random disturbance, and *i* and *j* indicate firm and country, respectively.

Besides the determinants of SMEs' survival probability which is the main (long‐term) performance indicator in our theoretical discussion, we also analyze two others *Short‐term Impacts* of COVID‐19 on firms—*Temporary Closure* and *Employment Decrease*—which represent dependent variables in our empirical model formalized in Equation (2):

(2) $$E\left(\text{Short}-{\text{term Impacts}}_{\mathrm{ij}}\right)=\begin{array}{l} \phi (\beta_{0}+\beta_{1}{\text{Innovation}}_{\mathrm{ij}}\\ +\;\beta_{2}{\text{Institutional Connectedness}}_{\mathrm{ij}}\\ +\;\beta_{3}{\text{Governance}}_{\mathrm{ij}}\\ +\;\beta_{4}\text{Management Experienc}e_{\mathrm{ij}}\\ +\;\beta_{5}\mathrm{No}\;\text{Financial Constrain}t_{\mathrm{ij}}\\ +\;\beta_{6}\text{Female}-\text{owne}d_{\mathrm{ij}}\\ +\;\beta_{7}{\text{Micro}}_{\mathrm{ij}}+\beta_{8}{\text{Small}}_{\mathrm{ij}}\\ +\;\beta_{9}\text{Firm}\;\mathrm{Ag}e_{\mathrm{ij}}\\ +\;\beta_{10}\text{Retai}l_{\mathrm{ij}}\\ +\;\beta_{11}{\text{Services}}_{\mathrm{ij}}\\ +\;\beta_{12}\text{Number of Suppor}{\mathrm{ts}}_{\mathrm{ij}}\\ +\;\varepsilon_{\mathrm{ij}}), \end{array}$$

where the notation is as in Equation (1).

As explained earlier, *Number of Supports* is treated as an endogenous variable and an instrumental variable (IV). The Probit estimator is used with two instruments—the legal status of the firm (incorporated or not) and the firm being a grant recipient pre‐COVID‐19. It is reasonable to assume that the firm's legal status and grant recipient status are exogenous to the impact of the pandemic on firms. Furthermore, to control for the potential heteroscedasticity, the Probit models—Equations (1) and (2) ‐ are estimated with robust standard errors.

## ESTIMATION RESULTS

### Which firms are likely to survive?

To investigate the partial impact of each explanatory variable of interest in relation to firm permanent closure, we estimate Equation (1) using Probit estimator. The estimation results are presented in Table 4. The findings from the full sample (column 1) are, generally, in support of our three main hypotheses because the coefficient signs are as expected. We find that *Innovativeness* and *Institutional Connectedness* have a significant impact on the survival of SMEs. However, neither the *Governance* variable nor the *Management Experience* variable is statistically significant. We further investigate the effect of *Governance* on survival and find that it is moderated by the firm's financial status (these results are available on request): for firms which are not financially constrained, *Governance* has the expected negative, significant effect on permanent closure.

**TABLE 4:** Permanent closure

	(1)	(2)	(3)	(4)
Variables	All countries	Old‐EU	New‐EU	Non‐EU
Innovativeness	−0.0100*** (0.0016)	−0.0224*** (0.0051)	−0.0066*** (0.0023)	−0.0055*** (0.0019)
Institutional connectedness	−0.0047** (0.0020)	−0.0085 (0.0053)	−0.0060** (0.0026)	−0.0049 (0.0036)
Governance	−0.0024 (0.0028)	−0.0151** (0.0073)	−0.0054 (0.0043)	−0.0013 (0.0043)
Management experience (Log)	−0.0020 (0.0034)	0.0218 (0.0195)	−0.0045 (0.0046)	−0.0087** (0.0042)
No financial constraint	−0.0143*** (0.0038)	−0.0140 (0.0108)	−0.0120** (0.0053)	−0.0120** (0.0059)
Female‐owned	0.0045 (0.0042)	−0.0038 (0.0130)	0.0031 (0.0057)	0.0117* (0.0062)
Micro	0.0170*** (0.0058)	−0.0021 (0.0181)	0.0125 (0.0085)	0.0124 (0.0090)
Small	−0.0041 (0.0048)	−0.0193 (0.0168)	−0.0087 (0.0073)	−0.0002 (0.0071)
Firm age (log)	−0.0097*** (0.0032)	−0.0368*** (0.0075)	−0.0030 (0.0049)	−0.0010 (0.0046)
Retail	−0.0134*** (0.0046)	−0.0266** (0.0112)	−0.0010 (0.0070)	−0.0174** (0.0069)
Services	−0.0059 (0.0043)	0.0051 (0.0130)	−0.0021 (0.0060)	−0.0096 (0.0067)
Observations	9572	1985	4255	3332

*Note*: Manufacturing and medium‐size firms are reference categories. Robust standard errors in parentheses;

*** *p* < 0.01,

** *p* < 0.05,

* *p* < 0.1.

We also find that all control variables are, generally, statistically significant and with the expected signs. Firms which are not financially constrained are less likely to close permanently, but micro firms are more likely to close permanently. Older firms are less likely to close permanently. Retail firms are less likely to close permanently relative to firms in other sectors.

Our analysis of subsamples—old‐EU (column 2), new‐EU (column 3), and non‐EU (column 4) firms—reveals interesting heterogeneity in the effects of our explanatory variables. The effect of *Innovativeness* holds in all subsamples. However, the *Institutional Connectedness* effect is statistically significant only in the new‐EU subsample, while the *Governance* effect is statistically significant only in the old‐EU subsample. The effect of *Management Experience* also differs across subsamples and is only statistically significant in the non‐EU subsample as expected. Thus, considering all the results so far, we argue that our three hypotheses are supported overall, conditional on relevant moderating institutional factors.

In terms of control variables, *No Financial Constraint* remains a significant factor in the new‐EU and non‐EU subsamples. However, there is no heterogeneity in the firm size effects, while firm age only matters in the old‐EU subsample: older firms are less likely to permanently close. Firm ownership shows a heterogeneous effect with female‐owned firms more likely to permanently close in the non‐EU subsample only. The retail sector firms are least likely to permanently close relative to other sector firms, across subsamples; however, the effect is not statistically significant in the new‐EU subsample.

### Short‐term impacts and the role of government support

The analysis in the previous section concerned the long‐term impact of COVID‐19. COVID‐19 also affects short‐term decisions concerning temporary closure and employment decrease. These strategies are driven by both pull and push factors, thus creating a heterogeneous pool of firms. On one hand, firms which are genuinely struggling for survival may temporarily suspend operations. On the other hand, unconstrained firms, which face exogeneous reduction in demand may, under the pressures of the government lockdown policy, temporarily suspend operation and benefit from the financial compensation available. In this case, the two temporary outcomes could be seen as strategic alternatives; if the company values its workforce more than revenue losses, conditional on resource availability, it would choose the temporary closure option. Alternatively, the choice could be reduction in the workforce (costs) while maintaining revenues from continued operation.

In this section, we estimate the partial effects of our three key variables, firm‐specific controls, and government support measures on *Temporary Closure* and *Employment Decrease* (working hours reduction or worker retrenchment) applying Equation (2) and using IV Probit estimator. Considering all the arguments above, it is likely that some of the expected effects may be obscured due to the variation of COVID‐19 policies across country subsamples.

The estimation results with temporary closure as the dependent variables are presented in Table 5. The findings from the full sample (column 1) are clearly supportive of our three main hypotheses because the coefficients are significant, and their signs are as expected. We find that *Innovativeness*, *Institutional Connectedness*, and *Governance* reduce temporary closure. *Management Experience* also has a statistically significant, negative effect as expected. All control variables are statistically significant and with the expected signs. Firms which are not financially constrained are less likely to temporarily close, but micro firms are more likely to temporarily close as also are female‐owned firms. Older firms are less likely to close. Retail and other service firms are more likely to close temporarily during the pandemic, relative to firms in manufacturing. Government support (the number of support instruments obtained) has a significant impact on reducing temporary closure.

**TABLE 5:** Temporary closure

	(1)	(2)	(3)	(4)
Variables	All countries	Old‐EU	New‐EU	Non‐EU
Innovativeness	−0.0055* (0.0031)	−0.0286* (0.0169)	−0.0043 (0.0040)	−0.0019 (0.0076)
Institutional connectedness	−0.0237*** (0.0051)	−0.1027*** (0.0310)	−0.0167*** (0.0061)	0.0225 (0.0170)
Governance	−0.0222*** (0.0074)	0.0033 (0.0349)	−0.0248** (0.0101)	−0.0683*** (0.0181)
Management experience (log)	−0.0223*** (0.0084)	0.0572 (0.0481)	−0.0406*** (0.0110)	−0.0077 (0.0186)
No financial constraint	−0.0661*** (0.0106)	−0.0389 (0.0472)	−0.0131 (0.0150)	−0.0734*** (0.0237)
Female‐owned	0.0442*** (0.0124)	−0.1053 (0.0800)	0.0529*** (0.0148)	0.0542*** (0.0202)
Micro	0.0427*** (0.0155)	0.0927 (0.0745)	0.0651*** (0.0208)	0.0261 (0.0362)
Small	0.0086 (0.0134)	0.0384 (0.0652)	0.0229 (0.0176)	0.0288 (0.0310)
Firm age (log)	−0.0531*** (0.0084)	0.0405 (0.0372)	−0.0127 (0.0122)	−0.0690*** (0.0193)
Retail	0.0315** (0.0145)	0.1189* (0.0614)	0.0992*** (0.0199)	−0.1028*** (0.0312)
Services	0.0750*** (0.0121)	−0.0674 (0.0813)	0.0948*** (0.0154)	0.0529* (0.0301)
Number of supports	−0.0812*** (0.0241)	0.7668*** (0.2274)	0.0095 (0.0294)	−0.4686*** (0.0919)
Observations	9243	1870	4132	3241
Wald‐test for exogeneity	44.61***	29.14***	8.01***	55.90***

*Note*: Manufacturing and medium‐size firms are reference categories. Robust standard errors in parentheses;

*** *p* < 0.01,

** *p* < 0.05,

* *p* < 0.1. The null hypothesis of no endogeneity is rejected (Wald test) for all specifications suggesting IV Probit is an appropriate estimator.

Analysis of subsamples—old‐EU (column 2), new‐EU (column 3), and non‐EU (column 4) firms—again reveals important heterogeneity in effects. The effect of *Innovativeness* is only statistically significant in the old‐EU subsamples. The *Institutional Connectedness* effect is statistically significant in both the old‐EU and the new‐EU subsamples, while the *Governance* effect is statistically significant in the new‐EU and non‐EU subsamples as we expected. The effect of *Management Experience* varies across subsamples, but it is only statistically significant in the new‐EU subsample. Overall, in this part of the analysis, we still find support for our three hypotheses, conditional on EU membership.

Considering control variables, *No Financial Constraint* is a significant factor in the non‐EU subsample. However, heterogeneity emerges in the effects of firm size and age—micro firms are most likely to temporarily close only in the new‐EU subsample, while older firms are less likely to temporarily close in the non‐EU subsample only. Female‐owned firms are more likely to temporarily close in both the new‐EU and non‐EU subsamples. We observe an interesting heterogeneity in the sectoral effects: retail firms in the non‐EU subsample are less likely to temporarily close while the opposite is likely in the old‐EU and the new‐EU subsamples. Other service firms are more likely to temporarily close compared to manufacturing firms, in both the new‐EU and non‐EU subsamples.

The effect of government support is quite heterogeneous across subsamples. In the old‐EU subsample, it increases the probability of temporary closure, while in the non‐EU subsample the effect is the opposite; in the new‐EU subsample, no statistically significant effect is observed. The likely reason for this heterogeneity is the dual nature of the temporary closure strategy, which is driven by pull and push factors. In the old‐EU countries, temporary closure was an important component of the COVID‐19 containment strategy, which was rigorously implemented in the southern EU countries such as Italy, Portugal, and Spain (OECD, 2020:86–88). Also, the positive association of government support measures with temporary closure can be taken as indicating better targeting of the firms most affected by COVID‐19. In support of the later argument is our finding (estimation results available on request) that for firms which are not financially constrained, government support reduced the probability of temporary closure. In the non‐EU subsample, government support as generally designed to act as a source of liquidity (OECD, 2020:23–27) and thus led to reduction in the probability of temporary closure.

The results of the estimations with employment decrease as the dependent variables are presented in Table 6. The coefficients from the full sample (column 1) have the expected signs. However, only the *Institutional Connectedness* coefficient has a statistically significant, negative impact on SMEs' employment decrease. The *Management Experience* variable is statistically significant with a negative sign as expected. Thus, we only find support for H2 and partial support for H3. Most control variables, however, are statistically significant and with the expected signs. Firms which are not financially constrained are less likely to decrease employment as also are the older firms, while female‐owned firms are more likely to decrease employment. Micro (and small) firms are less likely to decrease employment which suggests that they place a relatively high value on their workforces. Retail firms are less likely to decrease employment, while other service firms are more likely to do so relative to firms in manufacturing. Government support has a significant, negative effect on employment reduction.

**TABLE 6:** Employment decrease

	(1)	(2)	(3)	(4)
Variables	All countries	Old‐EU	New‐EU	Non‐EU
Innovativeness	−0.0043 (0.0032)	−0.0299 (0.0196)	−0.0079 (0.0051)	−0.0035 (0.0060)
Institutional connectedness	−0.0093* (0.0052)	−0.0909** (0.0373)	−0.0104 (0.0076)	0.0255* (0.0134)
Governance	−0.0032 (0.0076)	0.0283 (0.0391)	0.0242* (0.0128)	−0.0452*** (0.0149)
Management experience (log)	−0.0207** (0.0088)	0.0322 (0.0572)	−0.0198 (0.0145)	0.0134 (0.0149)
No financial constraint	−0.0395*** (0.0111)	−0.0192 (0.0510)	−0.0677*** (0.0191)	−0.0260*** (0.0109)
Female manager	0.0345*** (0.0129)	−0.1393 (0.0911)	0.0341* (0.0196)	0.0588** (0.0236)
Micro	−0.0714*** (0.0159)	0.0127 (0.0791)	−0.0882*** (0.0262)	−0.0410 (0.0283)
Small	−0.0470*** (0.0139)	0.0371 (0.0709)	−0.0694*** (0.0230)	−0.0128 (0.0243)
Firm age (log)	−0.0393*** (0.0089)	0.0577 (0.0406)	−0.0223 (0.0156)	−0.0347** (0.0156)
Retail	−0.0534*** (0.0148)	−0.0425 (0.0637)	−0.1006*** (0.0237)	−0.0489** (0.0240)
Other services	0.0262** (0.0123)	−0.1516 (0.0948)	−0.0088 (0.0192)	0.0376 (0.0234)
Number of supports received	−0.0551** (0.0254)	0.8186*** (0.2939)	−0.1221*** (0.0382)	−0.2276*** (0.0779)
Observations	9243	1870	4132	3241
Wald‐test for exogeneity	18.66***	19.73***	26.03***	15.33***

*Note*: Manufacturing and medium‐size firms are reference categories. Robust standard errors in parentheses;

*** *p* < 0.01,

** *p* < 0.05,

* *p* < 0.1. The null hypothesis of no endogeneity is rejected (Wald test) for all specifications suggesting IV Probit is an appropriate estimator.

Subsample analysis—old‐EU (column 2), new‐EU (column 3), and non‐EU (column 4) firms—again reveals important heterogeneity in effects. However, the effect of *Innovativeness* is not statistically significant in any of the subsamples. The *Institutional Connectedness* effect is statistically significant, negative in the old‐EU subsample but positive in the non‐EU subsample. It seems that institutionally connected firms have the legitimacy to decrease employment if this is beneficial to them or because they can secure access to financial compensation for their employees affected by lay‐off or reduction in hours. The *Governance* effect is statistically significant, negative in the non‐EU subsample as we expected; however, the effect is positive in the new‐EU subsample. As previously, we find in additional estimations (results available on request) that the effect is especially associated with financially constrained firms. This suggests that *Governance* prioritizes investor interests. The effect of *Management Experience* does not vary across subsamples. Overall, with respect to the employment decrease strategy, we find mixed evidence in support of our three hypotheses, conditional on EU membership.

Considering control variables, *No Financial Constraint* is a significant factor in the new‐EU and non‐EU subsamples. However, heterogeneity emerges in the effects of firm size and age—micro and small firms are less likely to decrease employment only in the new‐EU subsample; older firms are less likely to decrease employment only in the non‐EU subsample. Female‐owned firms are more likely to decrease employment in both the new‐EU and non‐EU subsamples. There is some heterogeneity in the sectoral effects; retail firms in the new‐EU and non‐EU subsamples are less likely to reduce employment.

The effect of government support, as before, is quite heterogeneous across subsamples. In the old‐EU subsample, it increases the probability of employment decrease, while in the new‐EU and non‐EU subsamples the effect is the opposite. The likely reason for this heterogeneity is the dual nature of employment decrease strategies, driven by pull and push factors. In the old‐EU countries, employment decrease, accompanied by financial support measures, has been central to COVID‐19 containment strategies, which have been rigorously implemented in Italy, Portugal, and Spain (OECD, 2020:86–88). Besides, the positive association of government support measures with employment decrease indicates better targeting of those firms most affected by the pandemic. We find (estimation results available on request) that for firms which are not financially constrained, government support reduces the probability of employment decrease. In the new‐EU and non‐EU subsamples, COVID‐19 containment strategies have been less systematic; related support measures have acted as wage subsidies (OECD, 2020:23–27), reducing the probability of employment decrease.

## DISCUSSION, CONCLUSIONS, AND LIMITATIONS

Our contribution has been to deploy and test hypotheses drawn from three bodies of theory to examine COVID‐19's impact on SMEs' survival: resource dependency theory and institutional theory's Business Systems and Varieties of Capitalism variants. These theories were shown to have considerable predictive power in relation to SME survival. We also contribute to the empirical evidence base of the theories discussed by adding “transitional” environment specificity and developing the SME context. We show that outcomes varied between our three groups of European countries.

Our findings support the theoretically posited positive effects of innovativeness, institutional connectedness, and governance capability on SMEs' survival during the pandemic. The empirical support from our full‐sample analysis for our hypotheses is robust in relation to both permanent and temporary closure, reflecting long‐ and short‐term outcomes, respectively. In relation to the hypotheses, support from the employment decrease analysis is more mixed. Employment decrease is a short‐term strategy, and one severely affected by wider COVID‐19 containment policies.

From our subsamples, we identify important differences in outcomes between EU member states and non‐EU countries, and enduring legacies of “transition” in the new‐EU countries. Legacy effects persist. However, across the different subsamples, strong innovativeness is a uniformly important positive factor militating towards long‐term survival. Institutional connectedness and governance factors appear more important in relation to short‐term strategy adoption. The effects are heterogeneous across subsamples with institutional connectedness playing a more important positive role in the EU member states. SME governance has an important role in both new‐EU and non‐EU countries. Sometimes, results are conditional on firm financial status.

Our work confirms the importance of firm‐specific characteristics such as financial status, size, and age for SMEs long‐ and short‐term survival. Associated with the firm resource view and highlighting the institutional variation in our sample is the finding that female‐owned SMEs are more likely to suffer closure, in general. This is even more so in the non‐EU and (to some degree) new‐EU subsamples.

The overall positive effects of government support measures on SMEs' short‐term outcomes—closure or employment decrease—are strongly confirmed. However, we discover interesting heterogeneity of effects across subsamples. Firms in the old‐EU subsample seem to have been affected by the wider (multi‐functional) package for COVID‐19 containment, of which government support to firms was an integral part. Given this, support measures appear to induce more closures and employment decreases. In the non‐EU subsample, government support measures appear to have been more of the standard type where the main aim is to ameliorate firm financial status; in this sense, support has fulfilled its role. The new‐EU subsample represents an intermediate case.

Our results generate many management implications and contribute evidence to ongoing debates in this journal. Across all the countries we examined, the importance of the nexus of innovativeness, institutional connectedness, governance, and management capability emerged as key to SMEs' capacity to survive the pandemic. Innovativeness was especially significant. SME managers will note that our innovativeness measure is based on both incremental product and process innovation, and radical innovation arising from vertical and institutional collaborations. Our findings are consistent with and extend those of Santamaria and Surroca (2011) writing in this journal, who found that vertical and institutional collaborations had positive impacts on both types of innovation, and improved performance, outside of the COVID‐19 context. Thus, these capacities are significant in both normal and external‐shock contexts and for European SMEs in different institutional environments. Managers may also note that within the European Union, institutional connectedness was important to SME survival. Our finding extends Flatten et al. (2011) and Ferreira and Franco's (2017) results on the role of strategic alliances in European SME performance across a wider range of countries. These are further lessons for SME managers: SMEs operating across our different country groups need to recognize the importance of the intra‐firm governance function's fit and interaction with inter‐organizational networks in the process of knowledge acquisition and innovation, also demonstrated in this journal (Fliaster & Sperber, 2020). We show that these considerations are relevant to SMEs and not only to top managers in larger companies.

Our study has limitations, some of which could be resolved in future research. Although the WBES database provides comprehensive firm‐level data, the possibility exists that some observations are omitted from final samples due to incomplete survey responses. Another potential limitation, due to the data collection design, derives from the binary nature of our dependent variables. The severity of COVID‐19's impact could be better captured when data on the number of days SMEs were temporarily closed and the number of employees who lost their jobs during the pandemic become available. Using more precise measures of the pandemic's severity would further improve the analysis. It would also be interesting to further investigate how different types of firm benefitted from different government support schemes, and what the channels of impact were.
